# A randomized phase 1 study of safety, tolerability, and pharmacokinetics of MK-1088, a novel dual adenosine receptor antagonist, in healthy adult participants

**DOI:** 10.1007/s10637-024-01462-y

**Published:** 2024-08-10

**Authors:** Pranav Gupta, Manash Chatterjee, Yeonil Kim, Kathleen Deschamps, Lieselotte Lemoine, Kristien Van Dyck, Catherine Zhou Matthews, Sylvie Rottey, Aubrey Stoch, Eseng Lai

**Affiliations:** 1 Merck & Co., Inc., Rahway, New Jersey USA; 2 MSD Europe Belgium SRL, Brussels, Belgium; 3 Ghent University Hospital, Ghent, Belgium

**Keywords:** MK-1088, A_2A_ receptors, A_2B_ receptors, Dual inhibition, Safety, Pharmacokinetics

## Abstract

This phase 1 first-in-human study evaluated the safety, tolerability, and pharmacokinetics of MK-1088, a novel, small-molecule dual inhibitor of adenosine A_2A_ and A_2B_ receptors. Healthy adult participants were enrolled in two panels (*n* = 8 each) and randomly assigned to receive MK-1088 (*n* = 6) or placebo (*n* = 2) orally in each of five treatment periods. Participants in panel A received single ascending doses of MK-1088 at 1, 10, 50, and 150 mg or placebo in a fasted or fed (50 mg only) state. Participants in panel B received MK-1088 at 3, 25, 100, and 224 mg or placebo in a fasted state. Primary objectives were to evaluate safety, tolerability, and plasma pharmacokinetics following a single dose of MK-1088. The secondary objective was to evaluate the effects of a high-fat meal on pharmacokinetics. All participants (*n* = 16) completed the study (median age: 33 years [range: 20–43]; all were male). Treatment-related adverse events (AEs) occurred in 1 of 6 (17%), 4 of 6 (67%), 4 of 6 (67%), and 2 of 6 (33%) participants after receiving MK-1088 at 3, 25, 100, and 224 mg, respectively. No serious AEs or deaths due to any cause occurred. MK-1088 was rapidly absorbed after a single dose; half-life was ~ 11 h in the 100–224 mg dose range. The target concentration at 12 h (> 0.3 µM) was exceeded at the 50-mg dose level. MK-1088 plasma pharmacokinetics increased dose proportionately. A high-fat meal did not significantly affect pharmacokinetics at the 50-mg dose. MK-1088 was well tolerated and demonstrated dose-proportional pharmacokinetic properties that were not affected by a high-fat meal.

**Supplementary Information:**

The online version contains supplementary material available at 10.1007/s10637-024-01462-y.

## Introduction

Adenosine is an important natural mediator of immune suppression, and an adenosine-rich tumor microenvironment may present a barrier for effective immunotherapy [1]. Adenosine levels in the tumor microenvironment can be orders of magnitude higher (approximately 100-fold) than those observed in normal tissues (approximately 100 nM) [2]. Under normal physiologic conditions, extracellular adenosine is balanced by cellular uptake, thus maintaining adenosine at very low levels in the tissue microenvironment [1]. Aberrant cancer cell growth and hypoxic conditions in the tumor microenvironment produce and actively sustain high levels of extracellular adenosine triphosphate and adenosine [1, 3]. Under hypoxic conditions in tumors and inflamed tissues, the ectonucleotidases CD39 and CD73 are upregulated on endothelial, stromal, and some solid tumor cells and on several subsets of immune cells, leading to dephosphorylation of adenosine triphosphate and subsequently increased extracellular adenosine levels [4, 5]. Agonism of adenosine A_2A_ receptors on T cells, natural killer cells, and macrophages by adenosine directly suppresses T-cell proliferation and production of proinflammatory cytokines [1]. Agonism of adenosine A_2A_ and A_2B_ receptors on activated myeloid cells leads to production of immunosuppressive factors, including cytokines that secondarily inhibit T-cell function [1]. Inhibition of A_2A_ receptors, and possibly dual inhibition of A_2A_ and A_2B_ receptors, may improve the antitumor effects of immunotherapy.

MK-1088 is a novel small molecule dual inhibitor of adenosine A_2A_ and A_2B_ receptors. In vitro, MK-1088 was highly selective for A_2A_ over other adenosine receptor subtypes A_1_ (267-fold) and A_3_ (863-fold). MK-1088 was also highly selective for A_2B_ over A_1_ (16-fold) and A_3_ (53-fold). In mouse tumor models, MK-1088 has shown antitumor activity when combined with a programmed cell death protein 1 (PD-1) inhibitor. Therefore, MK-1088 could be used in combination with immunotherapies, such as pembrolizumab, to target immune escape mechanisms in solid tumors. Preclinical toxicologic evaluation of MK-1088 also supported further studies of MK-1088 in humans. Dual inhibition of the adenosine A_2A_ and A_2B_ receptors may block T-cell inhibition mediated by high levels of adenosine in the tumor microenvironment through two unique pathways. In this study, we assessed the safety, tolerability, and pharmacokinetic properties of MK-1088 to expand the understanding of this novel small-molecule drug to inform future studies in patients with solid tumors.

## Materials and methods

### Study design and participants

This was a randomized, placebo-controlled, double-blind, single-site, multiperiod, phase 1, first-in-human study. Healthy men and women of non–child-bearing potential aged between 18 and 45 years with a body mass index of  ≤ 32 kg/m^2^ and normal renal function (creatinine clearance > 80 mL/min based on the Cockcroft-Gault equation) were eligible for enrollment. Participants with a history of any malignancy, positivity for hepatitis B, hepatitis C, or HIV, or who require any prescription or nonprescription medications were ineligible.

Participants were enrolled in two panels (A and B). Four single-dose escalations of MK-1088 over five treatment periods were planned in each panel (Online Resource 1). Participants received study interventions in all periods under fasted conditions except for panel A, period 5, in which participants received MK-1088 or placebo with a high-fat meal. Specifically, participants in panel A received single ascending doses of MK-1088 at 1 mg, 10 mg, 50 mg, and 150 mg or placebo orally in a fasted state in periods 1–4 and 50 mg or placebo orally with a high-fat meal in period 5. Participants in panel B received single ascending doses of MK-1088 at 3 mg, 25 mg, 100 mg, and 224 mg or placebo orally in a fasted state in periods 1–4. Within each panel, participants were randomly assigned to receive MK-1088 or placebo in each of the periods. The assigned treatment for periods 3 and 5 (fasted/fed) in panel A were the same for each participant, such that the same participants received the active drug (MK-1088 50 mg) or matching placebo in both treatment periods. There was an interval of ≥ 3 days before dose escalation between panels and a washout period of ≥ 7 days between doses within each panel. A pharmacokinetic break for all participants occurred after completion of the 25-mg dose in panel B (period 2) and the 100-mg dose in panel B (period 3) to support dose escalation decisions.

### Objectives

The primary objectives of this study were to evaluate the safety, tolerability, and pharmacokinetic properties of single ascending doses of MK-1088. The primary pharmacokinetic hypothesis was that the true geometric mean concentration of MK-1088 at 12 h post dose (C_12h_) was > 0.3 µM at one or more well-tolerated fasted dose levels, based on predicted target engagement levels > 98% for A_2A_ receptors and > 80% for A_2B_ receptors. The aspirational target C_12h_ was 1 µM based on predicted target engagement levels > 99.5% for A_2A_ and > 90.0% for A_2B_. The secondary objective was to evaluate the effect of a high-fat meal on plasma pharmacokinetics compared with the fasted state.

### Procedures

Safety and tolerability were assessed by vital signs, 12-lead electrocardiogram (ECG), laboratory safety tests, and physical/neurological examinations as well as by reported adverse events (AEs), serious AEs, and other reportable safety events collected using open-ended, nonleading verbal questions. Safety was monitored from treatment allocation through 14 days after the last dose of study treatment. AE terms were taken from the Medical Dictionary for Regulatory Activities (MedDRA) version 24.0. Blood samples for pharmacokinetic analyses were collected before dosing and at 0.5 h, 1 h, 2 h, 4 h, 8 h, 10 h, 12 h, 24 h, 48 h, and 72 h post dose. The pharmacokinetic parameters analyzed were MK-1088 plasma concentration, area under the concentration-time curve (AUC) extrapolated to infinity (AUC_0–inf_), AUC from 0 to 24 h (AUC_024h_), time to maximum plasma concentration (T_max_), maximum plasma concentration (C_max_), concentration at 12 and 24 h post-dose (C_12h_ and C_24h_, respectively), clearance or apparent total clearance after oral administration (CL/F), apparent volume of distribution after oral administration (V_z_/F), and apparent terminal half-life (t_½_).

### Statistical analysis

Safety and tolerability were reported as summary statistics and were assessed in all participants who received ≥ 1 dose of study drug. Pharmacokinetic analyses were performed in all participants who completed treatment per protocol. The primary hypothesis that the geometric mean C_12h_ of MK-1088 exceeds 0.3 µM at one or more well-tolerated fasted doses was tested using a linear mixed effects model. Bayesian methodology was used for decision making, and posterior probability was calculated using model-based estimates. The posterior probability that the true geometric mean C_12h_ is > 0.3 µM was calculated for each dose level, assuming normality and a noninformative prior. The pharmacokinetic hypothesis would be satisfied by posterior probability of ≥ 60% for at least one dose level that also demonstrates an acceptable safety and tolerability profile. Assuming the true coefficient of variance is 30%, there was a 93% probability to yield ≥ 80% posterior probability that the geometric mean of C_12h_ was > 0.3 µM when the true geometric mean of C_12h_ was 0.4 µM (*n* = 6) under the univariate normal likelihood model with a noninformative prior.

Pharmacokinetic parameters were calculated by noncompartmental analysis using Phoenix WinNonlin^®^ Professional version 8.1 (Certara, Inc., New Jersey, NJ, USA). Model-based estimates for individual values of AUC_0–inf_, AUC_0–24h_, C_max_, C_12h_, C_24h_, CL/F, and V_z_/F at each fasted and fed dose level were natural log-transformed and evaluated with a linear mixed effects model containing fixed effects for treatment and a random effect for participants. An unstructured covariance matrix was used to allow for unequal treatment variances and to model the correlation between the treatment measurements for each participant (SAS v9.4, Cary, NC, USA). The Kenward and Roger method was used to calculate the denominator degrees of freedom for the fixed effects. 95% confidence intervals for the least squares means for each factor level were constructed on the natural logarithmic scale and referenced the t-distribution. Exponentiating the least squares means and lower and upper limits of these confidence intervals yielded estimates for the population geometric means and confidence intervals about the geometric means on the original scale. Dose proportionality of MK-1088 AUC_0–inf_, AUC_0–24h_, and C_max_ in the fasted stated was explored using a linear mixed effect model with natural logarithm of the dose, panel, and natural logarithm of dose by panel interaction as fixed effects and participants within panel as a random effect. The effect of food on MK-1088 pharmacokinetics was estimated using a geometric mean ratio of the fed state over the fasted state and 90% confidence intervals using the same linear mixed effects model.

## Results

### Participants

A total of 16 participants (8 in each panel) were enrolled and randomly assigned to receive either a single ascending dose of MK-1088 (*n* = 6) or placebo (*n* = 2) in each of the five treatment periods, except for panel B, period 5, which was cancelled (Online Resource 1). All participants completed the study. All participants enrolled were White males, and the median age was 33 years (range: 20–43) in panel A and 33 years (range: 21–38) in panel B (Online Resource 2).

### Safety

Across all periods, AEs occurred in 13 of 16 (81%) participants who received MK-1088 and 7 of 16 (44%) participants who received placebo (Table 1). All AEs were mild to moderate in severity, and there were no serious AEs or deaths due to any cause. The most common (≥ 10%) AEs in participants who received MK-1088, regardless of attribution to study treatment, were headache (5 of 16 participants; 31%), insomnia (4 of 16 participants; 25%), and abdominal pain, diarrhea, dysgeusia, dyspepsia, musculoskeletal stiffness, and postural dizziness (2 of 16 participants; 13% each). The most common AEs in participants who received placebo, regardless of attribution to study treatment, were headache and nasopharyngitis (2 of 16 participants; 13% each). Treatment-related AEs occurred in 7 of 16 (44%) participants who received MK-1088 and 2 of 16 (13%) participants who received placebo (Table 1). Treatment-related AEs occurred in 1 of 6 (17%), 4 of 6 (67%), 4 of 6 (67%), and 2 of 6 (33%) participants after receiving MK-1088 at 3, 25, 100, and 224 mg, respectively (Table 1). The most common treatment-related AEs were insomnia (38%), dysgeusia (25%), blurred vision (13%), and headache (13%) with MK-1088 and headache (25%) with placebo.

**Table 1:** Summary of adverse events

Treatment	*n*	Any AE, *n* (%)	Treatment-related AEs, *n* (%)
MK-1088	16^a^	13 (81)	7 (44)
1 mg	6	3 (50)	0 (0)
3 mg	6	4 (67)	1 (17)
10 mg	6	2 (33)	0 (0)
25 mg	6	5 (83)	4 (67)
50 mg	6	2 (33)	0 (0)
50 mg (fed)^b^	6	0 (0)	0 (0)
100 mg	6	6 (100)	4 (67)
150 mg	6	1 (17)	0 (0)
224 mg	6	4 (67)	2 (33)
Placebo	16^a^	7 (44)	2 (13)

*AE* adverse event

^a^In each of the two treatment panels, 8 participants were randomly assigned to receive MK-1088 (*n* = 6) or placebo (*n* = 2) per dose level in each treatment period; across all periods, all 16 participants received at least one dose of MK-1088 and all 16 participants received at least one dose of placebo

^b^Participants received a standard high-fat meal before MK-1088 dose

Dose-dependent increases in blood pressure and heart rate were observed over the 10-mg to 224-mg dose range with maximum mean increases from baseline of approximately 13 mmHg in systolic blood pressure (12 h post dose at the 224-mg dose level), approximately 13 mmHg in diastolic blood pressure (4 h post dose at the 150-mg dose level), and approximately 13 bpm in heart rate (12 h post dose at the 224-mg dose level). Notably, one participant had marked increases in systolic blood pressure of approximately 42 mmHg and heart rate of approximately 45 beats per minute from pre-dose baseline values at approximately 8.5 h post dose, following a single dose of 224 mg MK-1088. The systolic blood pressure and heart rate increases in this participant were transient in nature, lasting for approximately 1 h, and returned to pre-dose baseline values by 48 h post dose; the participant had no clinical symptoms. One participant who received MK-1088 25 mg had a rash (erythema and red macules), considered to be related to the study treatment by the investigator, that was mild in severity and was not associated with any local or systemic symptoms. The rash was localized to the chest and abdomen, did not spread or change over time, and resolved without intervention within 24 h. No clinically meaningful effects for study intervention were observed for laboratory safety tests, vital signs, or ECGs.

### Pharmacokinetics

The minimum target C_12h_ > 0.3 µM was exceeded at the 50-mg dose level of MK-1088 (model-based geometric mean 0.348 µM) with a posterior probability of 94.8% (Online Resource 3); thus, the pharmacokinetic hypothesis (≥ 60% posterior probability for at least one dose level) was satisfied. The aspirational target C_12h_ ≥ 1 µM was achieved at the 150-mg dose level (model-based geometric mean 1.204 µM) (Online Resource 3). Geometric mean for C_12h_ was > 0.3 µM at dose levels of 50 mg to 224 mg and was ≥ 1 µM at the 150-mg and 224-mg dose levels; geometric mean for C_24h_ was ≥ 1 µM at the 224-mg dose level (Table 2).

**Table 2:** Pharmacokinetic parameter values of MK-1088 in plasma following a single oral dose of MK-1088

Dose	*n*	C_max_, µM	T_max_,^a^ h	C_12h_, µM	C_24h_, µM	AUC_0–24h_, h*µM	AUC_0–inf_,^b^ h*µM	t_1/2_, h	CL/F, L/h	Vz/F, L
1 mg	6	0.0282 (12.2)	2.0 (1.0–2.0)	0.00872 (23.1)	NC^c^	0.255 (28.9)	0.310 (33.1)	6.88 (37.7)	6.89 (33.1)	68.3 (10.8)
3 mg	6	0.0853 (33.9)	2.0 (1.0–2.0)	0.0213 (64.0)	NC^c^	0.793 (47.9)	0.890 (58.2)	6.69 (43.0)	7.20 (58.2)	69.4 (18.3)
10 mg	6	0.225 (91.4)	2.0 (2.0–10.0)	0.0676 (43.4)	0.0302 (46.5)	2.28 (52.7)	3.00 (24.9)	11.0 (82.1)	7.11 (24.9)	113 (102.3)
25 mg	6	0.705 (31.1)	2.0 (1.0–2.0)	0.157 (54.3)	0.0602 (89.3)	6.10 (41.8)	6.87 (48.0)	7.99 (31.3)	7.77 (48.0)	89.5 (18.5)
50 mg	6	1.22 (45.4)	2.0 (2.0–4.0)	0.340 (60.5)	0.162 (96.0)	11.6 (44.7)	13.9 (47.0)	8.50 (32.7)	7.65 (47.0)	93.9 (41.7)
50 mg (fed)^d^	6	1.15 (11.5)	4.0 (4.0–4.0)	0.394 (36.3)	0.175 (73.6)	11.6 (25.0)	14.0 (31.6)	8.92 (15.1)	7.63 (31.6)	98.2 (16.9)
100 mg	6	2.13 (44.8)	2.0 (2.0–4.0)	0.787 (68.4)	0.459 (63.7)	24.6 (48.7)	31.8 (51.8)	11.2 (16.1)	6.72 (51.8)	108 (37.8)
150 mg	6	2.88 (20.8)	4.0 (2.0–4.0)	1.18 (38.6)	0.590 (84.4)	34.6 (24.8)	45.7 (37.9)	11.0 (30.9)	7.01 (37.9)	111 (8.2)
224 mg	6	4.74 (13.7)	4.0 (2.0–4.0)	2.03 (39.4)	1.03 (56.0)	55.2 (28.7)	71.4 (37.8)	10.6 (22.3)	6.70 (37.8)	103 (24.7)

All data presented as geometric mean (% geometric coefficient of variation) unless noted otherwise

*AUC*_*0–24h*_ area under the curve from 0 to 24 h; *AUC*_*0–inf*_ area under the concentration-time curve extrapolated to infinity; *C*_*12h*_ concentration at 12 h; *C*_*24h*_ concentration at 24 h; *CL/F* clearance or apparent total clearance after oral administration; *C*_*max*_ maximum concentration; *NC* not calculated; *t*_*1/2*_ apparent terminal half-life; *T*_*max*_ time to maximum plasma concentration; *Vz/F* apparent volume of distribution after nonintravenous administration

^a^Data are reported as median (minimum–maximum)

^b^For three participants (2 participants at 1 mg and 1 participant at 10 mg), the AUC extrapolated as a percentage of the total was > 25%

^c^Concentration values were below the limit of quantification at 24 h, therefore the geometric mean for C_24h_ was not calculated

^d^Participants received a standard high-fat meal before MK-1088 dose

**Fig. 1:**
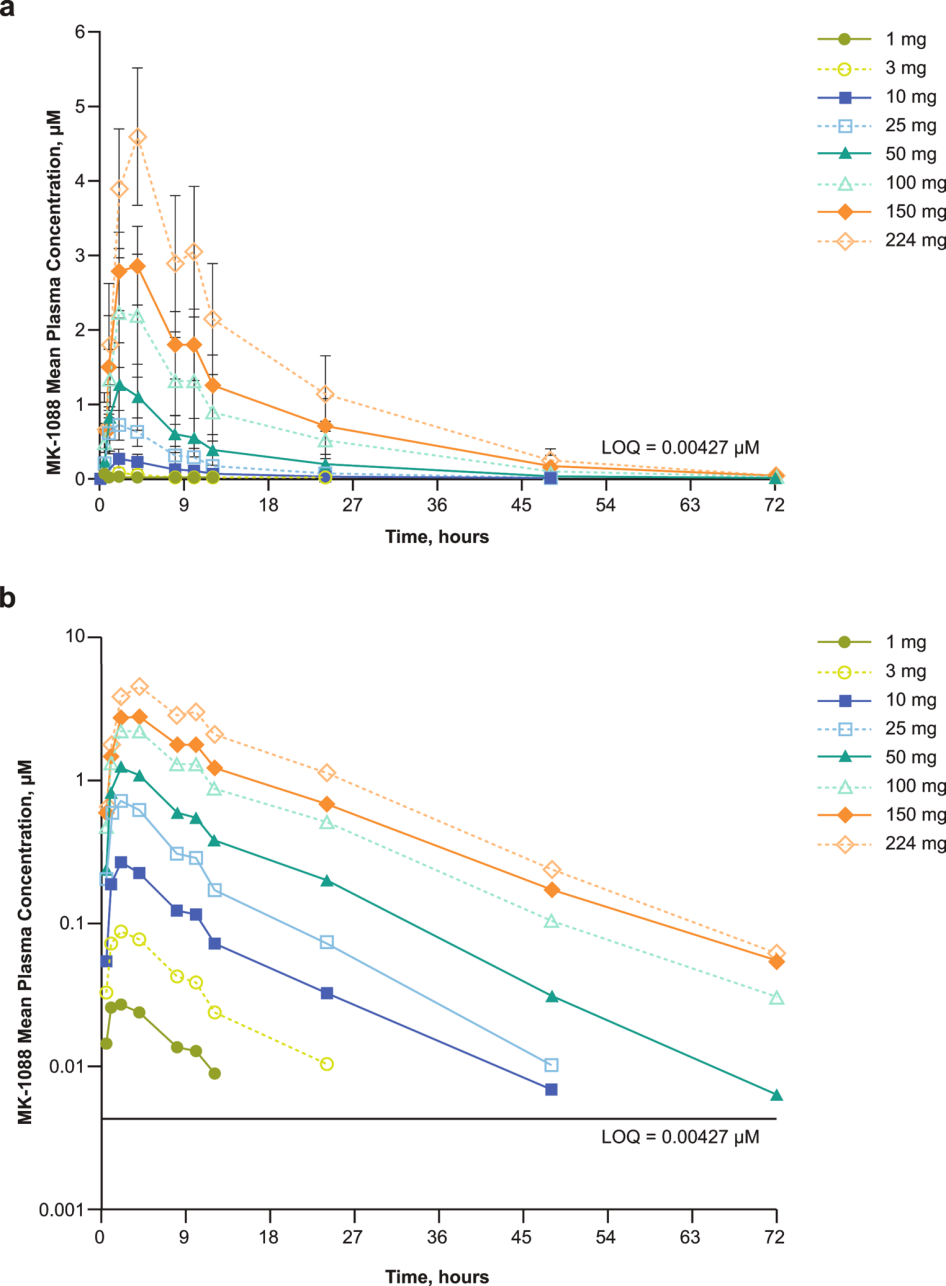
Plasma concentration of MK-1088 versus time following a single oral dose of MK-1088 on (**a**) a linear scale and (**b**) a semi-logarithmic scale. *LOQ* level of quantitation

MK-1088 was rapidly absorbed following single oral doses of 1 mg to 224 mg, with a median T_max_ of 2.0 to 4.0 h (Table 2; Fig. 1). The apparent terminal t_½_ ranged from approximately 6 to 11 h across the dose range tested and was approximately 11 h at the 10-mg dose and in the 100-mg to 224-mg dose range (Table 2). Plasma exposure of MK-1088 was approximately dose proportional over the tested dose range with slope estimates close to 1 for AUC_0–inf_, AUC_0–24h_, and C_max_ (Online Resource 4).

**Fig. 2:**
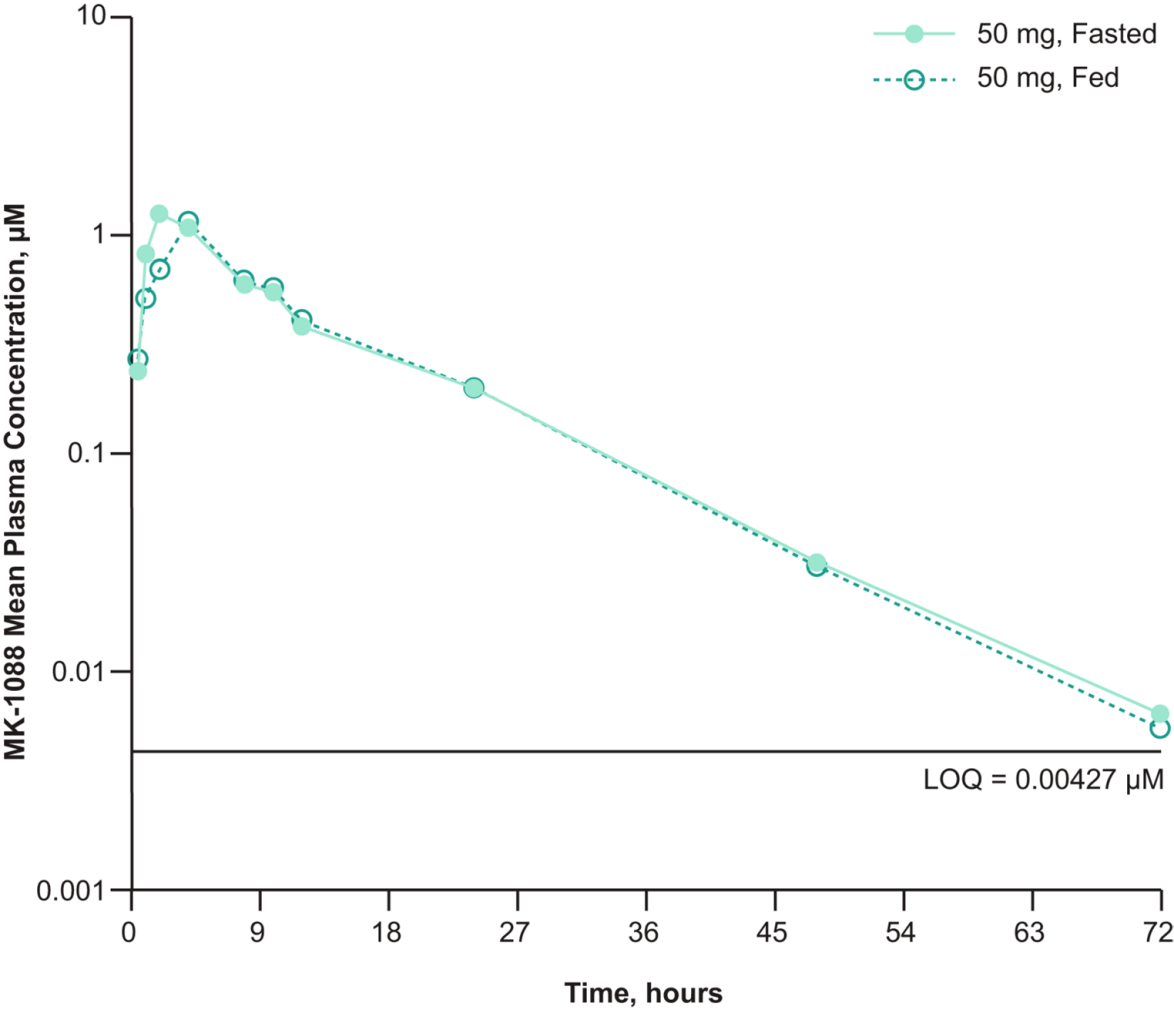
Plasma concentration of MK-1088 versus time following a single oral dose of 50 mg in the fasted and fed state on a semi-logarithmic scale. *LOQ* level of quantitation

Administration of MK-1088 50 mg after a high-fat meal delayed absorption compared with administration in the fasted state (median T_max_: 4.0 vs. 2.0 h; Table 2). However, administration in the fed versus fasted state had minimal impact on AUC_0–inf_, AUC_0–24 h_, or C_max_, with geometric mean ratios of 1.0, 1.0, and 0.94, respectively (Table 3; Fig. 2).

**Table 3:** Effects of a high-fat meal on plasma pharmacokinetics of MK-1088

	MK-1088 50 mg (fed)^a^ *n* = 6	MK-1088 50 mg (fasted) *n* = 6
**AUC**_**0–inf**_, h*µM		
Geometric mean (95% CI)	13.98 (9.64–20.26)	13.95 (9.62–20.22)
Geometric mean ratio^b^ (90% CI)	1.00 (0.72–1.40)	
**AUC**_**0–24h**_, h*µM		
Geometric mean (95% CI)	11.59 (8.35–16.09)	11.56 (8.33–16.04)
Geometric mean ratio^b^ (90% CI)	1.00 (0.70–1.44)	
**C**_**max**_, **µM**		
Geometric mean (95% CI)	1.15 (0.86–1.54)	1.22 (0.92–1.63)
Geometric mean ratio^b^ (90% CI)	0.94 (0.60–1.48)	
**T**_**max**_, **h**		
Median (min–max)	4.00 (4.00–4.00)	2.0 (2.00–4.00)

*AUC*_*0–24h*_ area under the curve from 0 to 24 h; *AUC*_*0–inf*_ area under the concentration-time curve extrapolated to infinity; *CI* confidence interval; *C*_*max*_ maximum concentration; *T*_*max*_ time to maximum plasma concentration

^a^Participants received a high-fat meal with their dose

^b^Geometric mean ratio is the exponentiated estimate of the mean of the natural log values obtained by a linear mixed effects model

## Discussion

This first-in-human phase 1 study evaluated the safety, tolerability, and pharmacokinetics of MK-1088, a novel dual antagonist of the adenosine A_2A_ and A_2B_ receptors, in healthy participants. The results show that single ascending doses of MK-1088 are well tolerated up to 224 mg in healthy male adults with no serious AEs or deaths observed. All AEs were mild to moderate. There were no clinically meaningful effects of study intervention on laboratory safety tests, vital signs, or ECGs. Target C_12h_ trough concentrations of MK-1088 were reached with doses of 50 mg and above when administered in the fasting state. MK-1088 demonstrated dose-proportional pharmacokinetic properties that were not meaningfully affected by a high-fat meal. Overall, the results from this phase 1 study indicate that MK-1088 is well tolerated and demonstrates a predictable pharmacokinetic profile. The safety and tolerability findings with MK-1088 in healthy volunteers prompted further evaluation of the antitumor activity of MK-1088 monotherapy and in combination with PD-1/L1 inhibitors in patients with solid tumors (NCT05394350).

Antagonism of the A_2A_ receptor with the vasodilation adenosine is expected to result in hemodynamic changes [6]. In line with this expectation, dose-dependent increases in blood pressure and heart rate were observed with MK-1088 over the 10-mg to 224-mg dose range. Although one participant had a marked increase in systolic blood pressure and heart rate after receiving MK-1088 at the 224-mg dose level that was transient in nature, these increases were not associated with clinical symptoms and returned to pre-dose baseline values by 48 h post dose.

The primary pharmacokinetic hypothesis of the study was satisfied; the minimum target C_12h_ of > 0.3 µM, based on the threshold predicted for target engagement of > 98% for A_2A_ receptors and > 80% for A_2B_ receptors, was achieved at the MK-1088 50-mg dose level. These predicted target engagement levels are expected to be associated with therapeutic efficacy. Moreover, the aspirational target concentration of ≥ 1 µM corresponding to projected target engagement levels of > 99.5% for the A_2A_ receptor and > 90.0% for the A_2B_ receptor was also achieved and maintained at 24 h at the 224-mg dose level, supporting a convenient once-daily dosing schedule. A high-fat meal delayed absorption but did not affect exposure at the MK-1088 50-mg dose level. MK-1088 plasma pharmacokinetic parameters were predictable and increased approximately dose proportionately, further supporting the feasibility of evaluating MK-1088 in patients with solid tumors.

MK-1088 is a novel investigational anti-cancer agent with a dual mechanism of action. A key strength of this study was that it was conducted in healthy participants, which offered the advantage of allowing rapid dose escalation into the likely therapeutic range for subsequent rapid evaluation in patients with malignancies. Evaluation in healthy participants allowed for more comprehensive and reliable measurement of pharmacokinetic parameters for thorough characterization of the MK-1088 pharmacokinetic profile. A limitation of the study is that it was not conducted in the intended target population of patients with a malignancy; thus, higher doses of MK-1088 that could possibly drive higher target engagement of A_2B_ receptor on myeloid cells could not be thoroughly evaluated due to risk-benefit considerations. Adenosine levels in the tumor microenvironment are several-fold higher than in healthy participants; therefore, the level of target engagement generated from a healthy participant study may be insufficient to maximize target engagement of both A_2A_ and A_2B_ receptors.

In conclusion, the dual A_2A_/A_2B_ receptor antagonist MK-1088 was generally well tolerated and demonstrated dose-proportional pharmacokinetic properties for single doses between 1 mg and 224 mg. Target concentrations predicted to provide therapeutic target engagement levels of A_2A_ and A_2B_ receptors were successfully attained at the 50-mg MK-1088 dose.

## Electronic Supplementary Material

Below is the link to the electronic supplementary material.

Supplementary Material 1

## Data Availability

Merck Sharp & Dohme LLC, a subsidiary of Merck & Co., Inc., Rahway, NJ, USA (MSD), is committed to providing qualified scientific researchers access to anonymized data and clinical study reports from the company’s clinical trials for the purpose of conducting legitimate scientific research. MSD is also obligated to protect the rights and privacy of trial participants and, as such, has a procedure in place for evaluating and fulfilling requests for sharing company clinical trial data with qualified external scientific researchers. The MSD data-sharing website (available at:http://engagezone.msd.com/ds_documentation.php) outlines the process and requirements for submitting a data request. Applications will be promptly assessed for completeness and policy compliance. Feasible requests will be reviewed by a committee of MSD subject-matter experts to assess the scientific validity of the request and the qualifications of the requestors. In line with data privacy legislation, submitters of approved requests must enter into a standard data-sharing agreement with MSD before data access is granted. Data will be made available for request after product approval in the USA and EU or after product development is discontinued. There are circumstances that may prevent MSD from sharing requested data, including country- or region-specific regulations. If the request is declined, it will be communicated to the investigator. Access to genetic or exploratory biomarker data requires a detailed, hypothesis-driven statistical analysis plan that is collaboratively developed by the requestor and MSD subject matter experts; after approval of the statistical analysis plan and execution of a data-sharing agreement, MSD will either perform the proposed analyses and share the results with the requestor or will construct biomarker covariates and add them to a file with clinical data that is uploaded to an analysis portal so that the requestor can perform the proposed analyses.
