# Supplementary material for: A randomized phase 1 study of safety, tolerability, and pharmacokinetics of MK-1088, a novel dual adenosine receptor antagonist, in healthy adult participants
Source: Invest New Drugs. 2024 Aug 10;42(5):492–9. doi: 10.1007/s10637-024-01462-y (PMC11625052; doi:10.1007/s10637-024-01462-y)
Supplement: Supplementary file 1 — Supplementary Material 1 [file 10637_2024_1462_MOESM1_ESM.pdf]

Title: A randomized phase 1 study of safety, tolerability, and pharmacokinetics of MK-1088, a novel dual adenosine receptor antagonist, in healthy adult participants

Target journal: *Investigational New Drugs*

Authors: Pranav Gupta[1], Manash Chatterjee[1], Yeonil Kim[1], Kathleen Deschamps[1], Lieselotte Lemoine[2], Kristien Van Dyck[2], Catherine Zhou Matthews[1], Sylvie Rottey[3], Aubrey Stoch[1], Eseng Lai[1]

Affiliations: [1]Merck & Co., Inc., Rahway, New Jersey, USA; [2]MSD Europe Belgium SRL, Brussels, Belgium; [3]Ghent University Hospital, Ghent, Belgium

Acknowledgements The authors extend their gratitude to all participants who took part in this study as well as to all investigators and site personnel. The authors also thank Steve Crowley, Pierre Daublain, Duane DeMong, Lyn Chen, Francheska Colon-Gonzalez, and Brad Roadcap at Merck & Co., Inc., Rahway, NJ, USA, for their contributions. Medical writing and/or editorial assistance was provided by Mehak Aggarwal, PharmD, of ApotheCom (Yardley, PA, USA). This assistance was funded by Merck Sharp & Dohme LLC, a subsidiary of Merck & Co., Inc., Rahway, NJ, USA.

Corresponding author:

Pranav Gupta

Email: pranav.gupta@merck.com

Online Resource 1 MK-1088 dosing schedule

Panel	Period 1		Period 2[c]			Period 3[c]			Period 4		Period 5	
**Panel A[a] (n = 8)**	1 mg		10 mg			50 mg			150 mg		50 mg[b]	
**Panel B[a] (n = 8)**		3 mg		25 mg			100 mg			224 mg		cancelled

[a]In each period, 6 participants received MK-1088 and 2 participants received placebo

[b]Dose given with a high-fat meal. The assigned treatment of periods 3 and 5 (fasted/fed) in panel A were the same for each participant such that the same participants received active drug of matching placebo in both treatment periods

[c]A pharmacokinetic break for all participants occurred after completion of the 25-mg dose in panel B (period 2) and the 100-mg dose in panel B (period 3)

Online Resource 2 Baseline characteristics

Baseline characteristic	Panel A	Panel B	Total
Male[a]	8 (100)	8 (100)	16 (100)
Age, median (range), years	33.0 (20–43)	33.0 (21–38)	33.0 (20–43)
Race[a]			
White	8 (100)	8 (100)	16 (100)
Ethnicity[a]			
Not Hispanic or Latino	8 (100)	7 (88)[b]	15 (94)

[a]Data are presented as *n* (%)

[b]Ethnicity was unknown for one participant in panel B

Online Resource 3 Model-based geometric mean C_{12h}

**Fasted dose**	**n**	**GM, µM (95% CI)[a]**	**Posterior probability (GM > 0.3 µM), %**
1 mg	6	0.008 (0.005–0.011)	0
3 mg	6	0.021 (0.015–0.030)	0
10 mg	6	0.072 (0.050–0.104)	0
25 mg	6	0.180 (0.125–0.260)	0.04
50 mg	6	0.348 (0.241–0.503)	94.8
100 mg	6	0.769 (0.532–1.110)	100
150 mg	6	1.204 (0.834–1.740)	100
224 mg	6	1.834 (1.270–2.650)	100

C_{12h} concentration at 12 hours; *CI* confidence interval; *GM* geometric mean

[a]Least-squares means and CIs were obtained by a linear mixed-effects model containing dose level and participants as fixed effects and a random effect, respectively

Online Resource 4 Assessment of dose proportionality of plasma MK-1088

	**Slope estimate[a] (95% CI)**	**Predicted fold-change[b] (95% CI)**
$AUC_{0–inf}$,[c] hr*µmol/L	1.01 (0.98–1.05)	239.4 (199.43–286.86)
$AUC_{0–24h}$,[c] hr*µmol/L	0.99 (0.94–1.03)	209.72 (161.89–263.48)
C_{max},[c] µmol/L	0.93 (0.88–0.99)	157.26 (118.54–209.21)

$AUC_{0–24h}$ area under the curve from 0 to 24 hours; $AUC_{0–inf}$ area under the concentration-time curve extrapolated to infinity; *CI* confidence interval; C_{max} maximum concentration

[a]Least squares estimates and CIs were obtained by a linear mixed model containing natural log of the dose as a fixed effect and participants within the panel as a random effect

[b]Expected fold-change with perfect dose proportionality, defined as a slope of 1.0, was 224. The predicted fold-change was calculated as the parameter at highest dose level over the lowest dose level

[c]Dose range from 1 mg to 224 mg (*n* = 6, each dose) administered in the fasted state was analyzed
